# The crisis that normalised time-shifting: Energy flexibility, price awareness and care during the energy crisis in Denmark

**DOI:** 10.1007/s12053-025-10327-z

**Published:** 2025-04-28

**Authors:** Kirsten Gram-Hanssen, Ander Rhiger Hanssen, Line Valdorff Madsen, Rikke Skovgaard Nielsen

**Affiliations:** Department of the Built Environment, Aalborg University, AC Meyers Vænge 15, 2450 Aalborg, Copenhagen SV Denmark

**Keywords:** Households’ practices, Energy flexibility, Energy crisis, Energy care, Energy vulnerability

## Abstract

The energy crisis during Winter 2022–2023 placed energy high on the public agenda in Denmark, including a hitherto unseen focus on dynamic electricity prices. This paper identifies a normalisation of following electricity prices online during the crisis and changing practices accordingly, the aim of the paper being to learn from this normalisation theoretically as well as policy-wise. The paper is based on a quantitative survey (N = 1000) and in-depth qualitative interviews (n = 30). Results showed that among those who indicate that they have flexible electricity prices almost 70% say that they follow prices daily, and an even larger share indicate that they are more aware of the timing of their electricity use compared to one year ago. Furthermore, more than half of the households used timers on their washing machines and dishwashers to time-shift appliance-use. Through an analysis of variation in interest and practices of time-shifting, we construct a parameter that we call ‘care for the energy system’, which showed more correlations than households’ socio-economy. The qualitative interviews revealed how experiences and engagement in time-shifting of practices for some households was prompted by tight economic constraints and for other households by care for energy and interest in green energy consumption. The results suggest that variable prices coupled with media and authority communication can encourage people to care for the energy system through time-shifting. However, as economic incentives can have social consequences, price signals should be coupled with financial support for vulnerable households.

**Supplementary Information:**

The online version contains supplementary material available at 10.1007/s12053-025-10327-z.

## Introduction

The future energy system will increasingly rely on decentralised renewable energy, with digitalisation contributing to aligning energy production and energy consumption as cost-effectively as possible. While energy consumption follows social rhythms with small peaks in the morning and larger peaks in the evening (Hansen et al., 2022; Trotta, 2020; Walker, 2014), the variable energy production from renewable energy follows weather changes in wind and sun. Time-shifting of energy-consuming practices in households, away from the peaks and towards times of high renewable production, thus becomes a crucial factor in the future sustainable energy system (Friis & Christensen, 2016; Parrish et al., 2020; Powells & Fell, 2019). However, time-shifting and load management were not widespread before the energy crisis. The purpose of this paper is to understand how, why and to what extent a normalisation of time-shifting took place, and what we can learn from it that can support increased time-shifting outside of times of crisis.

Aligning energy production and demand can include combinations of automation and different types of dynamic pricing which incentivise consumers to time-shift their consumption (Hansen, Harboe, and Lund 2021; Parrish et al., 2020; Smale et al., 2017). Dynamic pricing includes fixed prices that follow the time of the day and is always higher in peak-hours, called time-of-use (ToU) prices, or dynamic prices that follow energy prices on the energy spot market relying on production and consumption forecast, called real-time pricing (RTP), or it can be a combination of these and other types of time-dependent incentives (Faruqui et al., 2017; Kessels et al., 2016).

The energy crisis 2022–2023 in Denmark, in some European countries called a cost-of-living-crisis, was sparked by the Russian war against Ukraine. It was followed by an uncertainty from authorities on the extent to which an actual scarcity of energy could emerge, thus leading Danish authorities to encourage households and others to save energy. During the summer and autumn 2022, electricity prices rose substantially, and real-time-pricing could vary dramatically within a few hours. The authorities’ announcement of a possible energy crisis and the increasing prices led to a huge public media interest in energy production and consumption. In September 2022, the five most downloaded apps were apps showing electricity consumption and the variable electricity prices the coming days.^1^ Thus, energy suddenly changed from being in the background of everyday life to becoming visible. The energy crisis became an interruption (Chappells & Trentmann, 2018) which at least for a certain time period put aside normality and led to changes in everyday practices.

Based on this understanding of disruptions, we expect that a situation such as the energy crisis can imply changes in energy practices, and that the impact of such changes varies depending on the households’ socio-demographic situation and their interest in the energy system. Our purpose is to understand how and why a normalisation happened, and what can be learned from it. In the following we will first present the most important and recent research on household’s time-shifting and flexibility. This is followed by an introduction to the theoretical framing of our analysis based on practice theory and the concept of care. The quantitative and qualitative methods of the study are then described, followed by an analysis similarly divided into a quantitative and a qualitative part. As will be further described in the method sections, the two empirical approaches did not ask precisely the same questions, which is why the analysis of the two datasets are presented in separate sections. In the conclusion, the findings from the two analyses are drawn together, presenting the combined results which then points to the main insights and recommendations from the overall study.

## Existing research on time-shifting and energy flexibility

Time-shifting and dynamic energy prices have been addressed by the social sciences in different ways. Trotta (200) has shown that there is little difference in the timing of peaks across different social groups, though the magnitude varies with socio-economical and building characteristics. Further, it has been shown that rather than using socioeconomics to explain differences in peak consumption, clustering in the timing of activities can show more distinct patterns which can shed light on which groups that might be advantaged or disadvantaged by an introduction of dynamic pricing (Torriti & Yunusov, 2020). This approach follows from focusing on the activities, or the practices, of what people do in their everyday life, rather than focusing on their energy consumption (Strengers, 2012; Torriti, 2017). Thus, following this logic, time-shifting of energy consumption is about changing the rhythm and timing (Walker, 2014) of the performance of different practice in everyday life.

Questions of energy justice following dynamic pricing has focused on winners and losers, arguing that the design of prices and programs is essential (Calver and Simcock 2021; Jalas and Numminen 2022). Studies have shown how families with children may be more constrained in timing their energy consuming practices, including how practices are linked together in sequences and synchronised with childcare institutions (Friis & Christensen, 2016; Nicholls and Strengers 2015). Furthermore, gender aspects have been identified as crucial for understanding the timing and shifting of energy consuming practices (Johnson, 2020; Strengers et al. 2022). The term “flexibility capital” has been introduced to account for the different factors affecting the possibility to consume in a flexible way, including working patterns, social practices and the household’s context of energy consumption (Powells & Fell, 2019).

Households’ responsiveness to dynamic prices has been found to vary greatly with the specific program (Crawley et al. 2021; Faruqui et al., 2017) as well as with the context, including types of electricity use (heating/cooling or only appliances), and presence at home during daytime, e.g. pensioners (Kessels et al., 2016). A study investigating the energy crisis 2022–2023 in Norway found that peak hour reduction was lower than the all-day average reduction (Hofmann and Lindberg 2024). The study attributes savings to a combination of prices and media discourse about the crisis, as there was not always a day-to-day correlation between price and time of use. Norway is a highly electrified society, with most homes being heated by electricity and almost 30% of households having electrical vehicles (EVs). 48% reported that they were time-shifting their use of dishwasher, washing machine and dryer, however, in households with electric heating this may have little effect on the total consumption (Hofmann and Lindberg 2024). Similarly, other studies have found that using dishwashers, washing machines and dryers are the most easily time-shifted activities (Friis & Christensen, 2016; Smale et al., 2017). The Norwegian study showed that households who reduced their peak hour consumption were those who declared that they followed the hour-to-hour prices online and had an automated charging agreement for their EV (Hofmann and Lindberg 2024). These results are in line with other studies showing that enabling technologies such as online price and consumption information as well as possibilities of automation increase the peak-hour reductions (Faruqui et al., 2017).

Research has also addressed the extent to which households reduce peak consumption based on price, technological possibilities or a broader concern and care for energy systems, society and environment. Strengers, for instance, describe how households express a sense of social responsibility when responding to peak price signals (Strengers 2010), whereas Christensen et all. (2020) find that aspects of doing something for the common good, including securing energy sovereignty, impact the extent to which households time-shift.

## Theoretical approach: care ethics and practice theory

Doing something for the common good in relation to the energy system has been linked to a notion of caring for energy systems (Chambers 2022; Damgaard et al., 2022). The notion of care relates to a feminist tradition of care ethics (Tronto, 2013), which originally focused on humans caring for other humans, building on human dependency and vulnerability, as a contrast to rationality and rights in traditional ethical approaches. Caring for nonhumans, nature and the environment has since been included in the caring concept (Mol, Moser, and Pols 2010; Puig de la bellacasa, 2017), leading to a focus on how humans, as well as non-humans, can be givers and receivers of care (Gram-Hanssen, 2024). In relation to energy systems, the care concept moves the focus from individual responsibility, rational choice and human rights towards a focus on relations, dependency, necessity and needs (Damgaard et al., 2022). Thus, saving or time-shifting energy use can be interpreted as caring for the energy system rather than as rationality or saving money. Caring for the energy system can include helping to prevent blackouts, securing enough energy for all, or preventing climate change.

Understanding the energy system in relation to care also includes seeing energy consumption as related to caring for oneself or one’s household. Residential consumption in general is strongly related to care (Godin & Langlois, 2021) as is energy consumption (e.g. for food and heating comfort). Such caring-related energy consuming practices are highly gendered (Sadowski, Strengers, and Kennedy 2021). Care and energy consumption thus can be based on a desire to care for one’s own household or to care for the energy system and the environment. This implies that a caring perspective on energy will include dilemmas and controversies in everyday practices, balancing between the needs for the common good with the needs of the household. In the acknowledgement of such dilemmas, care ethics as a theory has the advantage compared to more rational and individualist approaches, that it can encompass such dilemmas (Gram-Hanssen, 2024).

Importantly, including care ethics in understanding energy consumption is not the same as saying that care ethic is a main element in people’s energy consuming everyday practices. Following a practice theoretical perspective means understanding consumption as moments in practices (Warde, 2005) and similarly to see energy consumption as something which follows from the performance of practices (Røpke & Christensen, 2012). Practices are here seen as collective doings and sayings held together by various elements such as rules, understandings, competencies, knowledge and engagements; named, used and combined in slightly different ways by different authors (Gram-Hanssen, 2011). Engagements and meanings in this understanding relates to the specific practice, e.g. the meaning of serving a proper meal or washing clothes. A caring ethic in relation to the energy systems, however, can be interpreted as what Schatzki (1996) called *general understandings* implying meanings and engagements which sits across many different practices and which may thus shape the specific performance of several different practices (Gram-Hanssen, 2021).

During the repetitive and ongoing performance of collective everyday practices, normality is established either in the general population or among specific groups. This normality works as a form of lock-in (Sahakian, 2018). Practices, however, do change. One way to understand this is that change happens when the elements holding them together change (Shove et al., 2012). Changes in elements holding practices together can for instance be the introduction of new technologies or products, e.g. new tariff structures or apps for visualising hourly electricity prices and consumption. Changes in practices can also follow from disruptions or crisis, where the normal performances and their daily rhythms of practice are temporally disrupted (Chappells & Trentmann, 2018), either by law as seen during the covid- 19 crisis (Greene et al., 2022) or by the physical breakdown of infrastructures of energy provision (Heidenstrøm & Kvarnlöf, 2018). The energy crisis in 2022 in Denmark did not represent a disruption in either a legal or physical sense. Rather the crisis implied a change in meanings associated with energy, following the sharply rising prices and for some households also “a physical-material limitation in the amount of energy that can be purchased due to financial constraints” (Gram-Hanssen et al., 2024:3).

## Methods

This paper uses a mixed-methods design that combines quantitative data from a survey questionnaire with qualitative data from an interview study. The mixed-method research design was convergent (Guest, 2013) in that the two methods address the same themes but have been gathered and analysed independently through a parallel mixed data analysis (Tashakkori & Teddlie, 2009). The qualitative and quantitative findings are integrated in the concluding discussion, making triangulation possible (Greene, 2007) i.e. to understand the same phenomenon through different methods to increase our combined understanding.

The two datasets were not designed to be directly comparable. They ask related but not identical questions. The survey was part of international collaborative work, where the focus was on energy saving during the energy crisis and to a lesser extent included flexibility. This implied that the survey only includes questions of flexibility related to practices of washing and dishwashing, whereas in the interviews, people address other practices related to time-shifting, such as cooking, entertainment and EV-charging. To allow both empirical materials to be analysed to their full potential, we present the quantitative and the qualitative analysis separately. The findings are subsequently combined in the concluding discussion. Nevertheless, the two datasets and their corresponding analyses support and add to each other, resulting in more strongly supported and broader overall findings.

### Quantitative data and methods

The quantitative data comes from a survey of adults (aged 18 or older) residing in Denmark at the time of data collection in March 2023.^2^ The questionnaire addressed experiences and reactions concerning the rapid increase of energy prices during 2022. Sampling was used to ensure an equal number of respondents per household income quintile, and responses were gathered by an agency using computer-assisted web interviews. Data collection stopped when 1,000 respondents were reached. The number of observations varies across variables as some questions depend on the responses on others.

The analysis uses a question battery, where respondents were asked to respond to a set of statements with the same response options from 1 “Strongly disagree” to 5 “Strongly agree”. The respondents were asked to which degree they agreed with a list of six statements ending the sentence “Saving energy is for me a way to…”. We conducted a factor analysis on the responses to the six statements. This revealed that the statements “…protest against the Russian war in Ukraine” and “…save money” had lower factor loadings than the others (below 0.600). The Cronbach’s Alpha increased when removing the two items. Table 1 shows the results of the final factor analysis with the factor loadings and scoring coefficients of the four items.

**Table 1:** Construction of the variable ‘energy caring’ (n = 1,000)

*Saving energy is for me a way to…*	Factor loading	Scoring coefficient	Uniqueness	KMO
… help Europe not having energy shortage	0.774	0.260	0.401	0.831
… help my local energy system to work more efficient	0.817	0.329	0.332	0.792
… help prevent blackouts	0.813	0.320	0.340	0.800
… help prevent climate change	0.657	0.163	0.568	0.887
*The factor ‘energy care’*				
Eigenvalue	2.359	Mean		0
Observations	1,000	Standard deviation		0.915
Cronbach's alpha	0.838	Minimum value		− 2.157
Kaiser–Meyer–Olkin measure (KMO)	0.821	Maximum value		1.502

A Kaiser–Meyer–Olkin measure of 0.821 suggests that there is an acceptable amount of common variance to construct a scale, and a Cronbach’s Alpha of 0.838 suggests a high level of internal consistency (Fabrigar & Wegener, 2012).

The items “… help my local energy system to work more efficiently” and “… help prevent blackouts” contributed the most to the underlying factor, whereas the items “… help Europe not having energy shortage” and “…help prevent climate change” contributed less. This indicates that the factor incorporates a local aspect. We have named the factor ‘energy caring’.

To analyse correlations between survey responses, we used three types of logistic regression models. When there were two responses, we used binary logistic regression. When the response variable had more than two outcome categories, but no specific order (as it contained the categories: “No”, “Don’t know”, and “Yes”), we used a multinomial regression model to estimate correlations. Multinomial regression models use a baseline category, which in this case was “No”, to estimate the probability of each of the other responses (“Don’t know” and “Yes”) being different from the baseline. Finally, when outcome variables had ordered response categories, ranging from 1 “Strongly disagree” to 5 “Strongly agree”, we used ordered logistic regression models (Wooldridge, 2010). The ordered logistic regression model assumes that a linear variable lies beneath the categorial responses (the proportional odds assumption). For all models, we interpreted the results using predicted probabilities.

### Qualitative data and methods

The qualitative data comprises 30 semi-structured interviews with households in Denmark, conducted during Winter 2022–23. They were, as the quantitative survey, centred on the experiences with and reactions to the energy crisis, but provided greater details on personal experiences, practices and explanations. The households were selected to mainly represent residents of homes heated by gas or electricity (rather than district heating), as they would have experienced the steepest energy price increases. This paper focuses on electricity use. For some interviewed households, this includes heating, but for many it does not. Anther paper from the same larger study deals explicitly with heating practices (Gram-Hanssen et al., 2024). The selection of households covers a diversity in socioeconomic characteristics, demographics and housing types. Furthermore, we were interested in interviewing households of primarily two types: disadvantaged households who had already been struggling prior to the increasing energy prices and inflation as well as households with an interest in energy and environmental sustainability. Recruitment was designed to ensure this. Two of the main sources for recruitment was thus a food waste and a green energy Facebook group. While the latter was efficient in recruiting energy-interested interviewees, getting in contact with disadvantaged households proved more difficult. A multitude of other recruitment channels was thus employed: personal networks, field trips and contacts in social housing organisations. Most interviews took place in the interviewee’s home and included a home tour to fertilise the talk on energy practices at home. Further information can be found in (Gram-Hanssen et al., 2024). The interviewees are presented in Table 2.

**Table 2:** Overview of interviewed households

Nr	Interviewee (pseudonym)	Housing type	Heating technology	Occupation	Household	Recruitment
1	Max and Anne	Terraced SH	Gas	Retired	2 adults	Area-based phone call
2	Elisa	Terraced SH	Gas	Retired	1 adult	Area-based phone call
3	Camilla	Terraced SH	Gas	Homecare, children with diagnosis	1 adult, 2 children	Through other interviewee
4 Phone	Marie	Detached OO	Gas	M in work W not in work (by choice)	2 adults	Local supermarket
5 Phone	Mette	Terraced SH	Gas	On social benefits	1 adult	Area-based phone call
6	Amelia	Terraced PR	HP	In work	1 adult	Personal network
7	Amy	Apartment SH	Gas	W on social benefits M in work	2 adults, 3 children	Café in social housing area
8 School	Muhammed	Apartment SH	Gas	In work	2 adults, 2 children	Language school
9	Connie	Terraced PR	Gas	Retired	1 adult	Personal network
10	Ella and Tom	Detached OO	Gas and wood stove	Retired	2 adults 1 child	Personal network
11	George and Vita	Detached OO	HP and gas	Retired	2 adults	Personal network
12	Sarah	Terraced SH	HP and electricity	On social benefits	1 adult	Facebook group for free items
13	Tara	Terraced PR	Electricity	On social benefits	1 adult	Facebook group for preventing food waste
14	Harry	Apartment SH	Gas	Retired	1 adult	Café in social housing area
15	Lily	Detached OO	Gas/ stove	On social benefits	1 adult, 2 children	Facebook group for preventing food waste
16	Vanessa	Detached OO	Gas	M in work W on parental leave	2 adults, 2 children	Facebook group for preventing food waste
17	Betty and Martin	Detached OO	Pellet stove	W Part-time employee M On social benefits	2 adults	Facebook group for preventing food waste
18	Caroline	Detached OO	Oil burner	In work	1 adult, 1 child	Personal network
19	Kent	Apartment OO	Gas	On social benefits	1 adult	Personal network
20	Joe and Rachel	Detached OO	HP	2 in work	2 adults, 3 children	Facebook group for green electricity
21	Nina and Jimmy	Detached OO	Electricity + HP	2 in work	2 adults, 2 children	Facebook group for green electricity
22	John and Elsa	Terraced OO	District heating	2 in work	2 adults 3 children	Facebook group for green electricity
23	Brian and Helen	Detached OO	Gas	M in work, W retired	2 adults	Facebook group for green electricity
24	Michael and Barbara	Detached OO	HP	2 in work	2 adults, 1 child	Facebook group for green electricity
25	Oscar and Roberta	Detached OO	Gas	2 retired	2 adults	Facebook group for green electricity
26	Sebastian and Tina	Detached OO	Solar heat + HP	2 in work	2 adults	Facebook group for green electricity
27	Dora and Josef	Allotment OO	District heating	2 retired	2 adults	Facebook group for minimalism
28	Maggie and Charles	Detached OO	Gas, just exchanged by HP	2 retired	2 adults	Facebook group for green electricity
29	Alex and Heidi	Detached OO	Gas	M in work, W on social benefits	2 adults, 1 child	Facebook group for green electricity
30	Vincent	Detached OO	Gas	2 in work	2 adults, 2 children	Facebook group for green electricity

## Quantitative analysis

Based on our study’s focus and the issues raised in the literature, the quantitative analysis is divided into two sections. First, the analysis focuses on how the energy crisis influenced the awareness of flexible electricity tariffs (not distinguishing between different types of dynamic pricing) and the engagement in changing the timing of electricity use. Second, the analysis addresses which practices the respondents tended to perform, exemplified by A) following electricity prices and using timers on B) dishwashers and C) washing machines. Both parts of the analysis investigate differences across levels of energy care and socio-demographic characteristics, such as economy, gender and age.

### Awareness of flexible tariffs and engagement in changing time-of-use

**Fig. 1:**
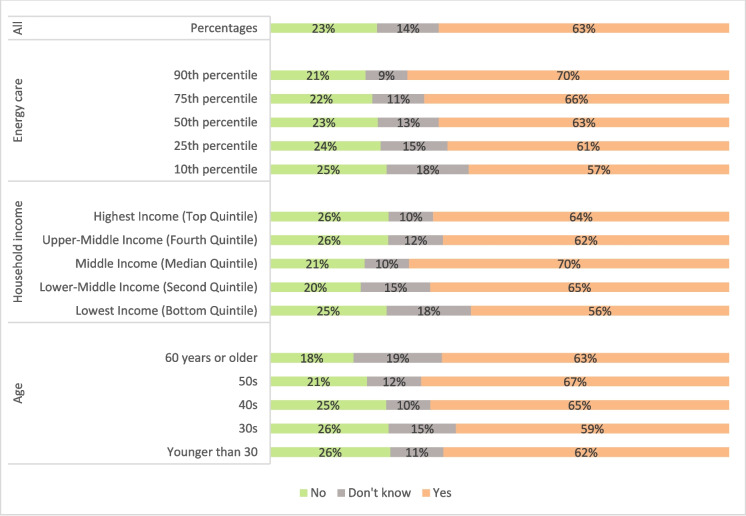
Predicted probabilities calculated from multinomial logistic regression of responses to the question: “Do you have flexible electricity tariffs, meaning that your electricity prices change during the day/week?” (N = 1,000). For the variables ‘Age’ and ‘Energy care’, the estimates are significant at a 90% level or higher, see Appendix 1

The respondents were asked if they had flexible electricity tariffs with the options of responding “No”, “Yes”, or “Don’t know”. To make the survey as simple as possible, this question did not differentiate between different types of tariffs, e.g. ToU or RT. The majority stated “Yes” (63.2%) while almost every fourth stated “No”. Crucially, we do not know whether the respondents actually had flexible tariffs. We only know what they think they had. However, it is fair to presume that practices are affected by the latter rather than the former. A multinomial logistic regression analysis showed significant differences across groups. Figure 1 shows selected predicted probabilities (see also Appendix 1). Based on these, three groups are more likely to state that they don’t know whether they have flexible electricity tariffs: those who are 60 + years, with a predicted probability of 19%, the lowest income group, with a predicted probability of 18%, and those who scored lowest on the energy care factor variable, with a predicted probability of 18%.

The highest predicted probability (70%) of stating to have flexible electricity tariffs is found for those who scored highest on the energy care factor variable, whereas the lowest is found for the lowest income group. For stating “No” to having flexible electricity tariffs, the highest predicted probability is found for the group younger than 30 years (26%), but this is not significantly different from the others, while the lowest predicted probability is found for the group of 60 + years.

**Fig. 2:**
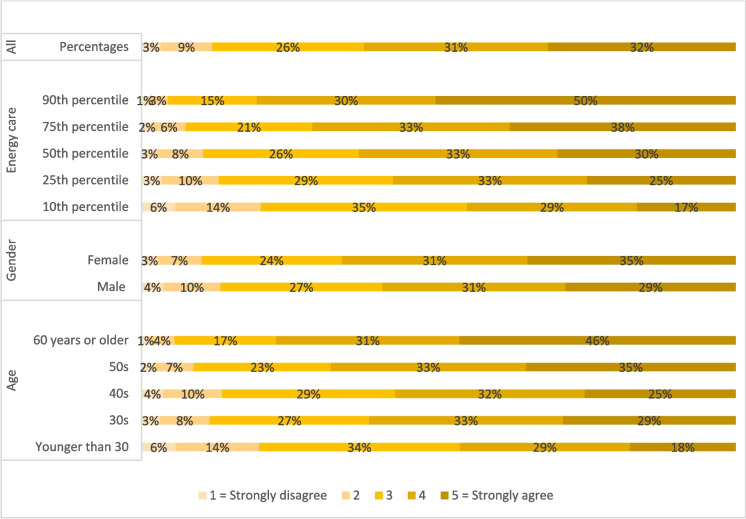
Predicted probabilities calculated from ordered logistic regression on the responses to the statement: “I do what I can to change in the time of use to adapt to variable energy prices” (N = 632). The selected variables have significant correlations at a 90% level or higher, see Appendix 2

Those stating “Yes” to having flexible electricity tariffs were presented with the statement: “I do what I can to change the time of use to adapt to variable energy prices”. 32% of the respondents strongly agreed with this (Fig. 2). Most respondents agreed with this statement, but with interesting variations.

Performing an ordered logistic regression on the five outcome categories reveals significant differences in responses when taking other variables into account (full model in Appendix 2). The predicted probabilities show that for those who scored very high on the energy care factor more than half strongly agreed. The group aged 60 + years followed with 47%. The lowest percentages for agreeing were found for the group younger than 30 years and those who scored lowest on the energy care variable. In addition, the ordered logistic regression shows that female respondents are more likely to agree with the statement of adapting to variable prices. This suggests that changing the time of energy use to adapt to variable energy prices is more likely done by women, the group of 60 + years, and those caring the most for the energy system. Surprisingly, the models suggest that household income is not linked to adapting to variable energy prices of awareness of timing of energy use.

**Fig. 3:**
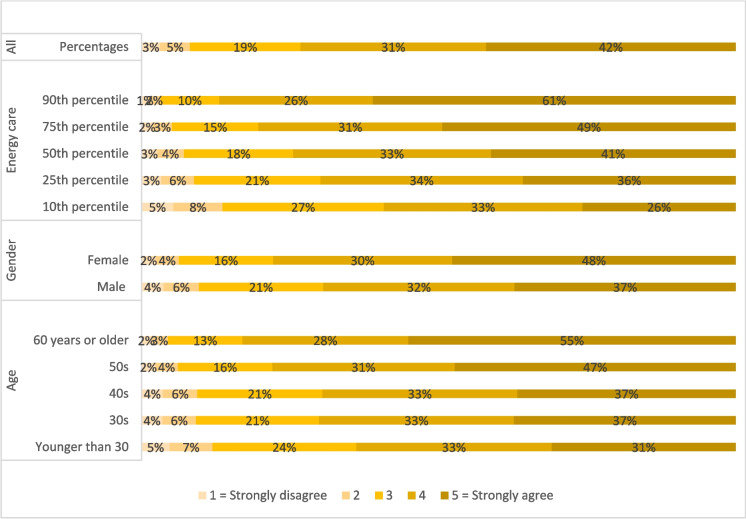
Predicted probabilities calculated from ordered logistic regression on the responses to the statement: “I am much more aware now compared to one year ago, about the timing of my electricity use?” (N = 632). The selected variables have significant correlations at a 90% level or higher, see Appendix 2

Respondents with flexible electricity tariffs were presented with a statement saying: “I am much more aware now compared to one year ago about the timing of my electricity use”. Figure 3 shows that almost half responded “Strongly agree” (42%), and very few disagreed with the statement. Still, there were substantial differences across groups. An ordered logistic regression showed that especially respondents who scored highest on the ‘Energy care’ factor were more likely to agree with the statement as was the group aged 60 + years and women.

### Using timers and following energy price variation

**Fig. 4:**
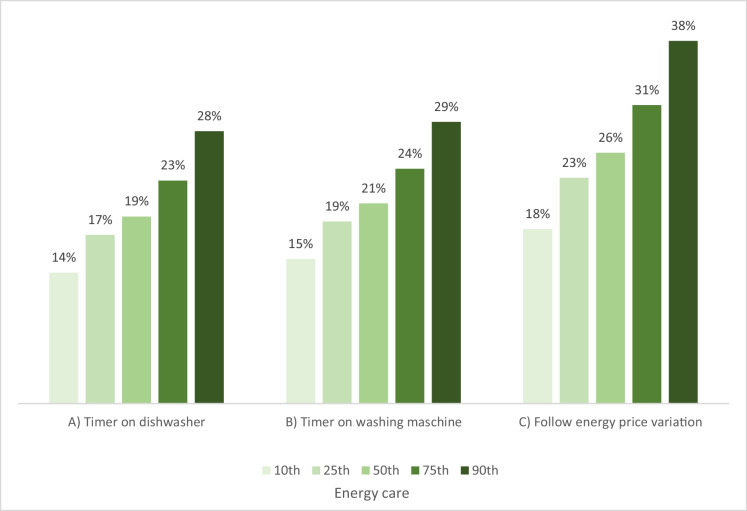
Predicted probabilities were calculated from logistic regressions on responding"Yes"to questions on using a timer for A) or B) or following price variation C) across'Energy care'percentiles, which variable estimate was significant in all three logistic regressions, see Appendix 3

**Fig. 5:**
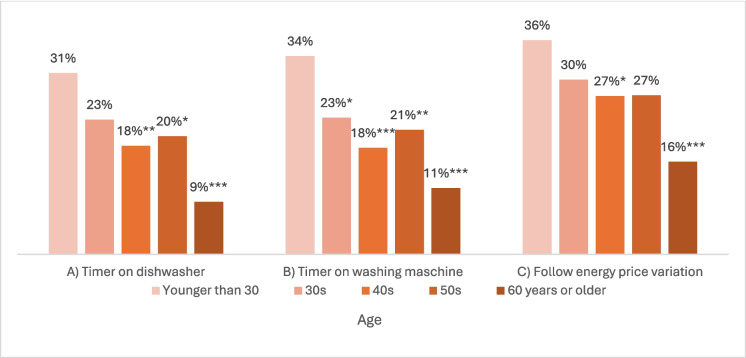
Predicted probabilities were calculated from logistic regressions on responding"Yes"to questions on using a timer for A) or B) or following price variation C) across age groups, * p < 0.10, ** p < 0.05, *** p < *0.01, significance based on logistic regression with “Younger than 30” as the reference group, see Appendix 3*

**Fig. 6:**
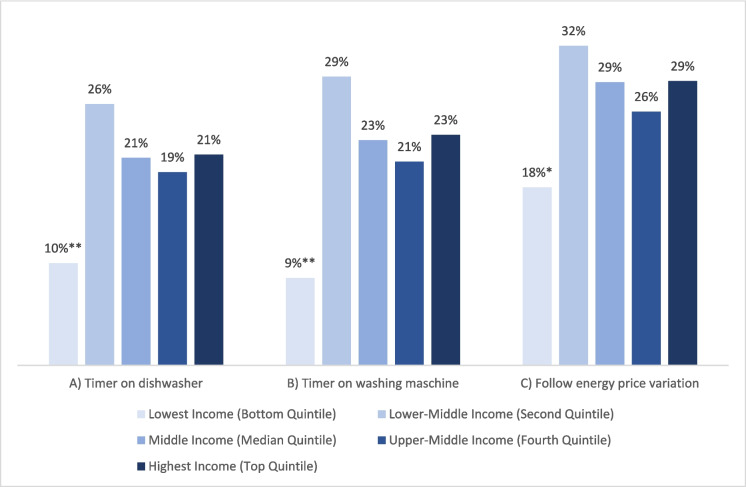
Predicted probabilities were calculated from logistic regressions on responding"Yes"to questions on using a timer for A) or B) or following price variation C) across income groups, significance based on logistic regression with “Lowest income” as the reference group, see Appendix 3

**Fig. 7:**
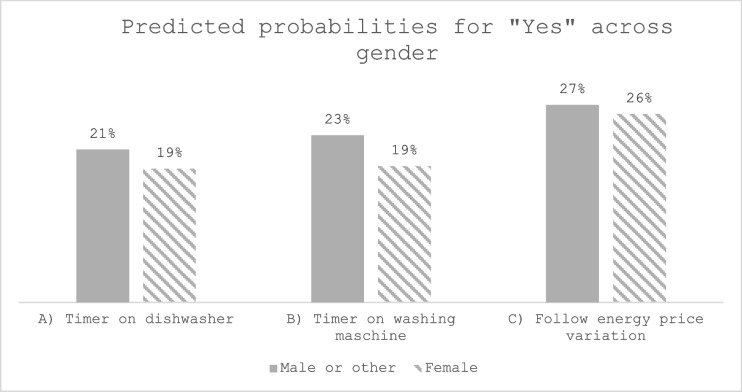
Predicted probabilities were calculated from logistic regressions on responding"Yes"to questions on using a timer for A) or B) or following price variation C) across gender, not any significant differences, see Appendix 3

The survey asked the question: “Do you use digital or smart technologies to handle the timing of your energy consumption?”. For those answering “Yes” (N = 359), three questions followed. They were asked which of three options applied to them: A) I use a timer on my dishwasher, B) I use a timer on my washing machine, and C) I use an electricity meter to know more precisely how much energy my appliances use. These three were recoded so that 0 included those that did not respond ‘Yes’ to the entry question (presented in Fig. 1). Figures 4 to 7 present predicted probabilities (marginal effects) based on Model i-iii (Appendix 3). Based on the significant differences in the logistic regression, these models indicate that the actions A), B) and C) are more likely to be performed more by those scoring higher on energy care and less by the respondents aged 60 + years and the lowest income group (Figs. 4, 5, and 6). Whereas Fig. 7 show no significance for gender.

## Qualitative analysis

Elaborating on the quantitative results on the normalisation of following energy prices, we first explore the interviews to understand variation in engagement of having apps and following energy prices on-line. Next, we focus on the time-shifting of practices, including which practice are time-shifted by which types of families as well as the meanings associated with this. Finally, the last section address the technological possibilities of timers and smart technology, specifically what impact these have on time-shifting, and how this varies with age and gender, as also shown in the quantitative analysis and discussed in the literature.

### Variation in having and using energy app’s

All interviewed households were aware of flexible prices and the possibility of time-shifting consumption, and all but two, to some extend followed energy prices and their own consumption online. One who did not, was an older woman, who declared that she did not have economic problems in paying her bills and thus did not care about it (#2). The other was a refugee, who just at the day of the interview had heard about the app showing variable prices. He was interested in trying to use it (#8). In all other households, at least one person had been looking at the variable prices regularly for some time. For the majority, this had started with the energy crisis and the media attention to this. Camilla explained that she had never looked at electricity prices before but started to do so this last summer. Asked whether they had a subscription with variable prices, she answered:

*Camilla*: Yes, we did, actually. I actually didn't know it until I... In fact, I didn't even have any idea who we had as electricity company. Well, I've never wondered about it. They send bills. It's probably about the same price everywhere.

*Interviewer*: So, when did you – this summer, or when did you start…?

*Camilla*: Yes. It was this summer.

*Interviewer*: And why? Because the bill came? Or because of the media talk?

*Camilla*: First it was media talk and then I could see that our bill had risen tremendously, so I thought, I just had to go in and find out then. (#3)

Like Camilla, many of the interviewees started to follow their electricity consumption, the variable prices or both at the time of the crisis due to a combination of media attention and noticing the rising prices. Tara (#13) expresses the normality of the app-use when saying: *"I actually think that all of us [ed: everyone] have looked at that app”*. Joe (#20) mentions, that besides the energy crisis, getting an electric vehicle made him more aware of the variable prices: charging the car is a huge electricity load, which is relatively easy to postpone to times with lower prices.

Some of the interviewees had apps or in other ways followed variable prices before the crisis. These interviewees generally see it as a hobby to follow their consumption and have a technical or environmental interest in it. Oscar has six different apps on electricity consumption data:

*Oscar*: Well, they represent them [the prices] a bit differently. And some of them are better at calculating the exact price. First, if we are talking electricity, then it is [name of electricity company] from which we get our electricity. They have an app. And it is excellent at showing yesterday's consumption. And then I also have one called"Min strøm"[My power]. That's also fine. I also talk to him quite a lot.

*Interviewer*: Oh, so you know him?

*Oscar*: No. I have made some suggestions to him on how he can make it better. Or if there are errors or something like that. I'm an old IT person, so I think it's a bit exciting how you can put something like this together. I like that very much. (#25).

Oscar is one of the more dedicated energy consumers among our interviewees. He explains how he every evening at 8 pm looks at the app to see their consumption of the previous day. Others, like Jimmy (#21), looks at the app every morning: *“I have my morning rituals”*. Following the amount of consumed energy is, however, not always related to smart meters and available apps. Brian (#23), for instance, explains how he used to read his meters of electricity, gas, and water every week and noting the figures in a spreadsheet. Following the consumption is a hobby to him, and now he does it through the app.

In contrast to the very dedicated and technically competent interviewees, others know about the apps and use them but find it difficult. We asked Tara (#13) if she can show us how she follows electricity prices on her phone:

*Tara*: Yes. Let me think. Then you have an option there and right now it costs such and such. And I'm not actually sure if it is with or without VAT. It is very difficult to understand, because in my view it is not clear enough whether it is with or without VAT. So, I think it is an approximate amount... And then...

*Interviewer*: But you use that ‘Watts’ one [an app] to follow the prices? You don’t use the ‘Andel Energi’ [name of electricity company] one for…?

*Tara*: No, because that one has been easier for me to figure out. (#13)

Others, like Dora and Josef (#27), experience problems related to the technical aspect of getting the app. Dora explains that her phone is old, so she cannot have many apps as it uses too much power. Instead, she checks it on her computer every morning. Doras’s husband, Josef, has a better phone and previously had the app installed, but then some months ago it required a new download, and he never did that: *“I'm probably a bit old-fashioned. I think I spend too much time on that PC. Including for my work and things like that”* (#27).

Despite these technical problems, most interviewed households were aware of variable electricity prices and followed them regularly. In the next section, we address whether this knowledge on varying prices led to time-shifting of energy consumption.

### Who is changing which practices and why?

Following the variable prices did not automatically lead to time-shifting of energy-consuming practices or knowing whether there was an economic benefit related to time-shifting. Some interviewees followed electricity prices regularly, but did not know whether their subscription was one with fixed or variable prices. Vanessa (#16) explains how they started time-shifting and only later realised that they had a flat rate subscription:

*Vanessa*: But I thought in the beginning that we had that thing [variable price agreement], and then I put a great deal of effort into...

*Interviewer*: So, you thought you had a price that varies throughout the day?

*Vanessa*: Yes, yes.

*Interviewer*: When did you find out that you didn’t have that?

*Vanessa*: When he [her husband] was tired of hearing the washing machine at night, he said, I'll just go in and check if it makes any difference. (#16)

Other interviewees were also aware of varying prices, but not sure what their own subscription was. Amelia (#6) gets in doubt during the interview about how it works. She says that she tries to time-shift part of her consumption, but she also explains, that she realised last week during a phone call with her electricity company, that she had a fixed price subscription. Only through the talk with us, she realises that even with her fixed price subscription there might be differences in price during the day, due to grid ToU tariffs. For these and other households, media attention and an increasing normalisation, where people talk about time-shifting as something normal to do, makes them time-shift despite not knowing their own potential for savings, or why they should time-shift.

When talking about time-shifting, all households mention dishwashers and washing machines. Some already used these appliances during nighttime before the crisis either for practical reasons or at least partly based on knowledge of the grid and electricity prices. Dishwashers seemed to be the appliance most often time-shifted. However, a few mentioned fears of fire or other hazards as a reason for not running appliances during the night. Amelia (#6) who instead runs it during the cheapest hours of the day, says that:

*Amelia*: I don't know. It's something about not having anything running when I'm sleeping. I know that you can easily do that, but I think it's just that... I don't like that. *(…)*. And maybe a little, then there is a bit of compulsive ideas. No, I do it when I'm awake.

While dishwashers seem to be the appliance considered easiest to time-shift, washing machines is a more recurring subject in the interviews. While the majority consider the possibility of time-shifting their washing, some do not do it, due to noise issues. Most people living in rented apartments or terraced housing explain that the housing association have rules against night-time washing. Some tenants have communal washing facilities, paying the same price per wash independent of time, and had to book time in advance. A few interviewees living in detached housing explain how bedrooms were too close by the washing machine for them to run it during the night. In addition, issues of when and where to hang the clothes are part of the considerations of when to wash and problems of planning ahead. Some interviewees in principle think that washing should be time-shifted, but find that it becomes too complicated:

*Alex*: And that's where it gets overcomplicated. So, then our everyday becomes...

*Heidi*: Laborious.

*Alex*: Then it gets too laborious somehow. That is, when we get there. It becomes too complicated. So, the power is there regardless.

*Heidi*: Especially regarding the washing machine. Then you might just wait. And now electricity became expensive again. Then you might just wait one more day, and suddenly, we are running out of clothes. Now we really have to wash, but then it would have been nice to be able to wash that one wash every day and not care about it.

*Alex*: And I think it's somehow fine that we do this. But when I hear you explain it, I think it is actually… It's too much. It is too complicated. We must also be able to live a reasonable life without...

*Heidi*: It gets stressful. (# 29)

Thus, time-shifting can be stressful for some households. Others, however, mostly the economically stressed, go further than time-shifting washing and dishwashing, including time-shifting cooking practices. Lilly, alone with two teenage sons, is one of them:

*Lilly*: I can make a stew for example [while it's cheap]. A stew also benefits from standing and brewing. We would actually... We use the oven as little as possible, really, as in really little.

*Interviewer*: Is this something new?

*Heidi*: And that's new from this summer. Where we said: okay, we have to light up the barbecue out there or something like that, if it should be other than on the frying pan.

*Interviewer*: So, you've used the barbecue too or what?

*Heidi*: Yes, yes. But now when [son’s name] came home, he loves lasagna. But then we had timed it, so that when it was cheap, we made the meat sauce and we made the béchamel sauce and then we could see that then it [electricity price] made that jump, but then luckily it was 7 o'clock, and then it went down to actually cheaper than earlier in the day, all of a sudden. Then it's just ready, so when it's five past seven or something, then we turn on the oven, and while it warms up, we assemble the lasagna and then in and then out again. (#15)

Some of the older interviewees, who had retired, had talked about the possibility of eating hot food for lunch and cold dinner, which was common in Denmark in the past, but had not actually done so. A few of the families with children explain that they can manage to do some of the cooking before the rising price at 5 pm, which suit them well as they like to eat early.

TV and computer use was mentioned in some interviews; again, mostly by the economically stressed households. Lilly (#15) explains how they do not turn on the television most evenings, and especially not those evenings with extreme prices. Instead, the family watches TV together on a smartphone. Similarly, Harry, a pensioner living on his own, has decided not to turn on his TV during the most expensive hours:

*Harry*: But my everyday life is then so that – to put it bluntly – after 5 pm, I don't turn on the TV. In the past, I have watched a lot of TV – yes, all sorts – but after 5 pm, I don't touch it, because then the rate increases. And if I must watch something, it's during the day. (#14)

While using a tablet or phone rather than a TV does not time-shift the practice of watching something, it lowers the consumption and time-shifts the energy use involved, as they charge the devices at less expensive times. In general, many of the interviewees reduce consumption in addition to time-shifting. Some households have unplugged their freezers, and in several households, teenagers’ use of gaming computers had been restricted. In these cases, there seemed to be a reasonable understanding of different appliances’ energy consumption and thus which appliances it makes sense to time-shift and reduce. In other cases, interviewees focus on time-shifting consumption types with very low loads, such as charging mobile phones. In contrast, heating, one of the largest loads, was only mentioned rarely by households with electric heating, despite research showing that most houses would be able to turn the heating off for some hours during peak time without experiencing comfort loss (Andersen et al. 2019).

### Relevance and implications of smart technology as enabler of flexibility

Having the possibility to time-shift requires availability of timers and smart control on appliances or being at home and awake at times of lower prices. As shown in previous studies there are differences in who has access to and thus can benefit from enabling technology (Powells & Fell, 2019). In the quantitative analyses we saw that people at the age of 60 + uses timers less than others despite being among those who time-shift the most. Our qualitative study elaborates on this. Elderly interviewees explain that it is easy for them to time-shift without enabling technologies because they, as pensioners, are home during daytime. A few interviewees explain that using washing machines during nighttime without a timer is not a problem. Caroline explains that being older means having to get up during the night anyways:

*Caroline*: No, but I can see how it is and then I can see that around 2 o'clock, for example, it is good. And it's pretty certain that I'm out peeing at 2 o'clock. So, if the machine is ready and the soap is in, then it's just that, even if I get out there half unconscious. So...

*Interviewer*: A timer is not needed?

*Caroline*: No, it’s not necessary at all. (#18)

Furthermore, across all age groups, several interviewees only became aware of having timers on their appliances at the onset of the energy crisis, whereas others realised that they did not have timers and were annoyed by this. Other types of smart control are also discussed in some interviews. Especially some of the most technically interested men talk about smart control, though some of them are not happy with what is available on the market, like Alex:

*Heidi*: You have also chosen a charger out there, which can communicate with the solar cells if the sun is shining and when it is cheapest on...

*Alex*: I had hoped so. It doesn't work.

*Heidi*: Oh, it doesn't? It was meant to...

*Alex*: It was meant to be able to. That's why I chose that charger, but that's also why I have that monitor out in the electricity meter, because then it was supposed to communicate with the charger and turn on the charger when the power was there. So, instead of it running into the neighbour, it would run into the car. It doesn't work, no. … (#29)

When the man of the household is interested in smart control, but the woman is responsible for some of the practices using electricity, this could result in discussions and conflict. As described in the literature (Johnson, 2020; Martin, 2022) there can be a discrepancy between the person interested in smart or demand control, and the person performing the affected household chores, and this discrepancy is often gendered. Alex and Heidi (#29) has such a discussion, where Heidi describes how she sometime has felt she was under electricity surveillance:

*Heidi*: And sometimes you also come home and say: Wow, we've used a lot of electricity.

*Alex*: Sometimes I come home and say that (laughs). And I haven't thought about it being a problem. It was just something I had noticed, and Heidi, she feels watched by me watching over her. When is electricity being used, how much electricity is used and whether it is used while the sun is shining. And it's a talk we've had.

*Heidi:* [...] So, then all of a sudden, I felt, like, electrically monitored.

Heidi and Alex reach an agreement that smart control and surveillance can be too much. In another household, while we are interviewing the spouses on similar subjects, the woman is clearly negatively affected emotionally, which the man does not seem to notice. Correspondingly, the literature has addressed the subject of policing other family members and the gender relations often connected to this (Martin, 2022).

In relation to energy vulnerable households some of the interviews illustrate how those who are at home during the daytime due to being unemployed, can benefit from using appliances in the cheaper hours of the day, thus not needing timers or smart technology. In one family, Lilly explains how washing more than one machine during the night cannot be done by using timers but requires her to get out of bed. She sets an alarm on her phone, if prices are low during the night:

*Lilly*: So, I can wash two machines of clothes during the night. Then I can set an alarm that says beep.

*Interviewer*: You get up and then… And go out and turn on the machine?

*Lilly*: Then I go out and change the clothes and then the bag [of wet washing] will have to be left until the next day.

*Interviewer*: Is this how you wash clothes, typically?

*Lilly*: It is very typical that it is washed at night, at least. Hardly any washing is done during the day. (#15)

Based on such issues of family conflicts and efforts of waking up during nighttime or other things which complicates everyday life, it becomes understandable why some households feel that flexible prices can be stressing, and that it can be a kind of freedom not to live by flexible pricing, maybe especially for those who are the most economically stressed:

*Amy*: I wouldn't want to have it like – some cook at 4 o'clock because it's cheaper than 6 o'clock, and if I feed my kids at 4, then they're hungry again and stuff like that. I don't want that. […] it's not a freedom, but... I might have thought more about it, if I saw that it cost me 6 DKK per kilowatt to cook at 6 o'clock versus 4 o'clock, then I might also have thought that maybe we should have it sooner or something, right. (#7)

Thus, for some interviewees, time-shifting is a balance between saving money and the sacrifices they feel time-shifting entail. For others, however, it is also a question of doing the right thing. Kent talks about a societal responsibility:

*Kent:* I try to be a little more aware of when things cost more. Not just for my own sake. But I think:"Okay, if that's where the highest load is, then it doesn't matter if I wash at 10 in the morning and 2 in the afternoon or in the middle of the night". Then I might as well think a little about also – I don't know if you can say it that way – the societal aspect and try to be a little more aware of when you have your consumption. (#19)

Similarly, Joe says that it can feel right to time-shift:

*Joe:* But in cases where it is made easy to shift, I think that is where we have jumped on it. Or relatively easy. Then I have no problem with being a bit first mover-like, if I can sense, that we can do it. It may well be that it may be a little more difficult, but in return you can have a clear conscience. (#20)

Thus, in some households, time-shifting is of limited trouble and result in positive feelings of caring for the energy system, society, or the environment. In some cases, though, this also involve an ongoing balancing between care for the family and care for the energy system, which in some families imply internal conflicts. And in yet other families, time-shifting is an economic necessity, which they can’t afford not to perform, in combination with other ways of lowering their expenses.

## Discussion and conclusion

During the energy crisis in Denmark 2022–23, we saw hugely increasing and variable prices on electricity together with a general attention in the media and the public discourse, including governmental campaigns. As shown in the analysis, this resulted in a normalisation of time-shifting. The respondents started to follow electricity prices online and to different degrees adapted their electricity consumption accordingly. Thus, more than 60% of the surveyed population agree that they do what they can to adapt to variable prices. This could be interpreted as an economically rational behaviour following the price signals. However, both the quantitative and the qualitative material suggest that there is more to it. From the quantitative analysis we learned that household income does not correlate with engagement in time-shifting energy use. From the qualitative interviews we heard that many people started to follow prices and adjust practices, without being aware of what tariff structures they had. Interviewees also expressed that it was not just about economy, but also about doing what everyone else did, as well as doing the right thing. Following the literature and theory, we have called this caring for the energy system.

Caring for the energy system was constructed as a factor from the survey results and included: wanting to help Europe not having energy shortage, helping the local energy system to work more efficiently, and preventing blackouts as well as helping to prevent climate change. Analysis showed that compared to socioeconomic background, this energy caring factor explained more variation in, 1) to what extend people was following energy prices and 2) to what extend they adjusted their consumption to the variable prices. Only the age group 60 + came out as positively correlated to questions of following prices and adjusting to them.

Gender differences did show up in the survey answers, in that females to a higher extent than males agreed that they are more aware now compared to one year ago on the timing of their electricity use, and that they do what they can to adjust their consumption to the variable prices. The qualitative analysis suggested potential gender related conflicts in time-shifting of practices as also described in the literature (Johnson, 2020; Martin, 2022). In some interviewed households, it was a specific interest or even hobby for the man to follow energy prices. However, some of the practices to be time-shifted were predominately done by the woman. One woman felt like she was under energy surveillance from her husband, which she did not like, even if she did express interest in caring for the energy system.

In general, washing clothes and dishwashing were the practices most often time shifted. If washing could easily be postponed or done in advance, most did so. From the survey we learned that those caring the most for energy were also those that used timers on appliances the most. One exception was the oldest age group 60 +, as they were the ones who had and used timers the least, despite other parts of both the qualitative and the quantitative analysis showing that this age group were among those who adjusted their time of use the most. The interviews can help explain this: interviewed pensioners explained that they could easily adjust to lower prices without using timers as they were home during daytime. Time-shifting practices without the use of enabling technologies is thus not necessarily a problem.

From the interviews we also heard that other energy consuming practices like cooking and media use could be time-shifted. However, this was mainly done in the most economically stressed households. Nevertheless, the only part of the quantitative analysis where income had a significant explanatory power, was whether people said that they had tariffs with variable prices. The poorest fifth of the population to a lesser degree stated that they had such tariffs. From the interviews we gained a possible explanation for this, from a mother of three in an economically stressed family. Having variable prices for her would have implied a daily stress to time-shift, because they had to worry about all their expenses. The more affluent families, in contrast, relayed satisfaction of being able to adjust, when doing so, and calmness when it was not possible or convenient.

From the theory we learned how disruptions in some of the elements holding a practice together can imply changes in practice – either temporarily or permanently. During the energy crisis, the meanings associated with energy use changed together with the rising prices and the public discourse about prices and issues of energy scarcity. Our analysis showed how these changes implied a normalisation in the Danish society in relation to following electricity prices on a daily basis and time-shifting mainly laundry and dish-washing practices. From the empirical data, quantitatively as well as qualitatively, we also learned, how caring for the energy system through time-shifting was weighed against other considerations, including care for oneself and household members, especially children. This way, the ability of care ethics, in contract to other abstract and objective theoretical ethics approaches, shows its advantage of being able to handle ambivalences in everyday life (Gram-Hanssen, 2024). This paper thus argues that care is an important concept for understanding when households are time-shifting, and when they are not.

To what extent this normalisation will last after the crisis, is outside the scope of this paper and the data it builds on. However, in supporting a future energy system where time-shifting in residential energy demand is needed, we can learn from this normalisation of time-shifting. First, it *is* possible to engage households in time-shifting. Price is part of this but not the only parameter that counts. Price is both a carrier of meaning related to what the energy system needs, and a carrier of meaning related to household economics. Thus, price and tariffs are highly relevant, but not only in an economic sense. Second, the media discourse and the way public authorities express what people can do to help society and help the energy system can have an impact on time-shifting. Many people wanted to time-shift, regardless of economic necessity. Our study thus identifies a potential to build on: Supporting the notion of time-shifting as a way to care for the energy system can give consumers meaning in their energy consuming practices. Public authorities and energy bodies can utilise this in their communication to increase time-shifting. Third, the same actors must however simultaneously be aware that economic incentives may have social consequences, which are crucial to be aware of in relation to gender and economy. As other studies have already suggested, there are possible gender dilemmas involved in time-shifting as many of the practices to time-shift are carried out by women, and this may put more stress on the female part of the household. Furthermore, there is an economic bias in how price signals work. Economically vulnerable households will be disproportionally hit by the increasing prices, leading to increased deprivation and stress. For affluent households, variable prices can be an incentive to do something a little better. Thus, two central policy recommendations are to support price signals with communication from authorities about the importance of time-shifting, building on people’s desire to care for the energy system, and to couple variable pricing with financial support for those who are disproportionally affected by price incentives.

### Limitations

Our study offers crucial insights based on a combination of quantitative and qualitative material. However, as all studies, it has its limitations. Survey sampling ensured an equal number of respondents per household income quintile which was essential as a key focus was the consequences for people of different financial situations. Nevertheless, the sample might still be skewed in unobserved ways. Furthermore, as mentioned previously, interviewees are asked about their perceptions which does not necessarily reflect facts e.g. in relation to whether they have flexible prices or not.

For the qualitative study, we have utilised a range of channels for recruiting disadvantaged households as these proved more difficult to reach. While this has mitigated the skewedness of our recruitment, the most disadvantaged or those struggling the most might not be represented as their surplus energy for taking part in a study might be limited. In both parts of the study, ethnic minority residents, particularly those of limited Danish language proficiency, are underrepresented; a well-known challenge when recruiting.

Finally, a limitation is that we did not have the possibility to combine survey and interview data with measured electricity consumption data. Such a combination would have been a strong data combination, though, is also difficult to achieve due to privacy and GDPR regulation (General Data Protections Rules) in obtaining access to household-based energy data.

## Supplementary Information

Below is the link to the electronic supplementary material.

Supplementary file1 (DOCX 38 KB)

## Data Availability

Anonymised qualitative data are available in research archive: https://zenodo.org/records/13847672 Anonymised quantitative data are available in research archive: https://zenodo.org/records/15163255

## Footnotes

1 The five most downloaded apps in Apple store was app’s about electricity: https://www.tv2fyn.dk/fyn/el-hitter-i-app-store-her-er-de-mest-populaere-apps-til-at-styre-dit-elforbrug

2 The survey was carried out in six different countries, which besides Denmark include Germany, Norway, The Netherlands, Switzerland and Polen. Only data from Denmark is included in this paper.
