# Supplementary material for: The crisis that normalised time-shifting: Energy flexibility, price awareness and care during the energy crisis in Denmark
Source: Energy Effic. 2025 Apr 28;18(5):39. doi: 10.1007/s12053-025-10327-z (PMC12034584; doi:10.1007/s12053-025-10327-z)
Supplement: Supplementary file 1 — Supplementary file1 (DOCX 38 KB) [file 12053_2025_10327_MOESM1_ESM.docx]

Appendix 1

*Multinomial logistic regression analysis on responding “Don’t know”, “Yes” or “No” (reference group) to the question: ”Do you have flexible electricity tariffs, meaning that your electricity prices change during the day/week?”.*

| Do you have flexible electricity tariffs, meaning that your electricity prices change during the day/week? | | | |
| --- | --- | --- | --- |
|  | No (Reference) | Don't know | Yes |
| Factor: Energy care |  | -0.210*  (0.122) | 0.162*  (0.088) |
| Household income |  |  |  |
| Lowest Income (Bottom Quintile) |  | 0.481  (0.363) | -0.437*  (0.262) |
| Lower-Middle Income (Second Quintile) |  | 0.514  (0.376) | -0.0331  (0.260) |
| Middle Income (Median Quintile) |  | Ref. | Ref. |
| Upper-Middle Income (Fourth Quintile) |  | 0.00129  (0.397) | -0.342  (0.247) |
| Highest Income (Top Quintile) |  | -0.225  (0.423) | -0.309  (0.247) |
| Gender |  |  |  |
| Male or other |  | Ref. | Ref. |
| Female |  | 0.0376  (0.236) | 0.155  (0.164) |
| Age | | |  |
| Younger than 30 |  | Ref. | Ref. |
| 30s |  | 0.277  (0.351) | -0.036  (0.239) |
| 40s |  | -0.155  (0.392) | 0.0844  (0.252) |
| 50s |  | 0.238  (0.374) | 0.293  (0.252) |
| 60 years or older |  | 0.930***  (0.355) | 0.366  (0.261) |
| Adults in household |  |  |  |
| One-adult |  | Ref. | Ref. |
| Two or more adults |  | -0.421  (0.259) | 0.0434  (0.195) |
| Children in household |  |  |  |
| No child |  | Ref. | Ref. |
| One or more children |  | -0.134  (0.287) | 0.0802  (0.187) |
| Housing tenure |  |  |  |
| Tenant |  | Ref. | Ref. |
| Homeowner |  | -0.905***  (0.295) | -0.0278  (0.193) |
| Housing type |  |  |  |
| Detached house |  | Ref. | Ref. |
| Semi-detached or terraced house |  | -0.335  (0.373) | 0.105  (0.236) |
| Apartment |  | 0.0108  (0.320) | -0.246  (0.220) |
| Location |  |  |  |
| Urban |  | Ref. | Ref. |
| Rural |  | 0.338  (0.279) | 0.00729  (0.198) |
| Peri-urban |  | -0.137  (0.363) | -0.247  (0.234) |
| Constant |  | -0.448  (0.516) | 1.078***  (0.357) |
| Observations | 1,000 | | |
| *Standardized beta coefficients; Standard errors in parentheses*  ** p < 0.10, ** p < 0.05, *** p < 0.01* | | | |

Appendix 2

*Ordered logistic regression analysis on responding from 1 “Strongly disagree” to 5 “Strongly agree” to the two statements in the table.*

|  | I do what I can to change the time of use to adapt to variable energy prices | I am much more aware now compared to one year ago, about the timing of my electricity use |
| --- | --- | --- |
| Factor: Energy care | 0.655^***^  (0.0893) | 0.605^***^  (0.0918) |
| Household income |  |  |
| Lowest Income (Bottom Quintile) | 0.0792  (0.252) | 0.215  (0.263) |
| Lower-Middle Income (Second Quintile) | 0.0589  (0.230) | 0.195  (0.237) |
| Middle Income (Median Quintile) | Ref. | Ref. |
| Upper-Middle Income (Fourth Quintile) | -0.237  (0.232) | 0.203  (0.236) |
| Highest Income (Top Quintile) | -0.139  (0.227) | -0.417  (0.227) |
| Gender |  |  |
| Male or other | Ref. | Ref. |
| Female | 0.340^*^  (0.156) | 0.490^**^  (0.159) |
| Age |  |  |
| Younger than 30 | Ref. | Ref. |
| 30s | 0.686^**^  (0.240) | 0.314  (0.244) |
| 40s | 0.486^*^  (0.247) | 0.286  (0.252) |
| 50s | 0.999^***^  (0.248) | 0.768^**^  (0.249) |
| 60 years or older | 1.512^***^  (0.257) | 1.117^***^  (0.258) |
| Adults in household |  |  |
| One-adult household | Ref. | Ref. |
| Two or more adults in the household | -0.0440  (0.193) | 0.311  (0.199) |
| Children in household |  |  |
| No child | Ref. | Ref. |
| One or more children | 0.262  (0.176) | 0.298  (0.183) |
| Housing tenure |  |  |
| Tenant or other | Ref. | Ref. |
| Homeowner | 0.184  (0.187) | -0.0382  (0.190) |
| Housing type |  |  |
| Detached house | Ref. | Ref. |
| Semi-detached or terraced house | -0.0150  (0.210) | -0.151  (0.215) |
| Apartment | -0.0882  (0.218) | -0.384  (0.223) |
| Location |  |  |
| Urban | Ref. | Ref. |
| Peri-urban | 0.327  (0.183) | 0.0665  (0.188) |
| Rural | 0.173  (0.232) | -0.0172  (0.233) |
| Cut point 1 | -2.605^***^  (0.397) | -2.750^***^  (0.406) |
| Cut point 2 | -1.130^**^  (0.351) | -1.694^***^  (0.365) |
| Cut point 3 | 0.569  (0.346) | -0.137  (0.349) |
| Cut point 4 | 2.079^***^  (0.355) | 1.388^***^  (0.354) |
| Observations | 632 | 632 |
| ** p < 0.10, ** p < 0.05, *** p < 0.01* | | |

Appendix 3

*Binary logistic regression analysis on responding “Yes” to doing the actions A), B) or C).*

|  | (A)  Using timer on dishwasher | (B)  Using timer on washing machine | (C)  Following energy price variation |
| --- | --- | --- | --- |
| Factor: Energy care | 0.386^***^  (0.0996) | 0.361^***^  (0.0968) | 0.412^***^  (0.0888) |
| Household income |  |  |  |
| Lowest Income (Bottom Quintile) | -0.889^**^  (0.338) | -1.178^***^  (0.348) | -0.641^*^  (0.274) |
| Lower-Middle Income (Second Quintile) | 0.330  (0.254) | 0.365  (0.245) | 0.186  (0.231) |
| Middle Income (Median Quintile) | Ref. | Ref. | Ref. |
| Upper-Middle Income (Fourth Quintile) | -0.0966  (0.258) | -0.138  (0.251) | -0.161  (0.233) |
| Highest Income (Top Quintile) | 0.0216  (0.252) | 0.0334  (0.244) | 0.00565  (0.228) |
| Gender |  |  |  |
| Male or other | Ref. | Ref. | Ref. |
| Female | -0.185  (0.176) | -0.287  (0.173) | -0.0709  (0.157) |
| Age |  |  |  |
| Younger than 30 | Ref. | Ref. | Ref. |
| 30s | -0.439  (0.242) | -0.569^*^  (0.240) | -0.326  (0.225) |
| 40s | -0.731^**^  (0.265) | -0.906^***^  (0.265) | -0.472^*^  (0.241) |
| 50s | -0.620^*^  (0.261) | -0.697^**^  (0.255) | -0.465  (0.237) |
| 60 years or older | -1.610^***^  (0.322) | -1.492^***^  (0.299) | -1.184^***^  (0.265) |
| Adults in household |  |  |  |
| One-adult household | Ref. | Ref. | Ref. |
| Two or more adults in the household | -0.0459  (0.218) | -0.00138  (0.214) | 0.271  (0.195) |
| Children in household |  |  |  |
| No child | Ref. | Ref. | Ref. |
| One or more children | **0.527^**^**  (0.195) | 0.432^*^  (0.193) | 0.307  (0.177) |
| Housing tenure |  |  |  |
| Tenant or other | Ref. | Ref. | Ref. |
| Homeowner | 0.217  (0.209) | -0.00298  (0.205) | 0.136  (0.187) |
| Housing type |  |  |  |
| Detached house | Ref. | Ref. | Ref. |
| Semi-detached or terraced house | -0.236  (0.244) | -0.343  (0.241) | -0.0860  (0.219) |
| Apartment | -0.436  (0.240) | **-0.611^**^**  (0.236) | -0.271  (0.214) |
| Location |  |  |  |
| Urban | Ref. | Ref. | Ref. |
| Peri-urban | -0.0132  (0.207) | -0.262  (0.206) | -0.140  (0.186) |
| Rural | 0.0517  (0.252) | -0.217  (0.251) | -0.0973  (0.231) |
| Constant | -0.794^*^  (0.375) | -0.263  (0.364) | -0.686^*^  (0.341) |
| Observations | 1,000 | 1,000 | 1,000 |
